# High Dietary Kuding Tea Extract Supplementation Induces Hepatic Xenobiotic-Metabolizing Enzymes—A 6-Week Feeding Study in Mice

**DOI:** 10.3390/nu12010040

**Published:** 2019-12-22

**Authors:** Svenja Wüpper, Alexandra Fischer, Kai Lüersen, Ralph Lucius, Hinako Okamoto, Yoshiyuki Ishida, Keiji Terao, Gerald Rimbach

**Affiliations:** 1 Institute of Human Nutrition and Food Science, University of Kiel, Hermann-Rodewald-Strasse 6, 24118 Kiel, Germany; fischer@foodsci.uni-kiel.de (A.F.); luersen@foodsci.uni-kiel.de (K.L.); rimbach@foodsci.uni-kiel.de (G.R.); 2 Anatomical Institute, University of Kiel, Otto-Hahn Platz 8, 24118 Kiel, Germany; rlucius@anat.uni-kiel.de; 3 CycloChem Bio Co., Ltd., 7-4-5 Minatojima-minamimachi, Chuo-ku, Kobe 650-0047, Japan; hinako.okamoto@cyclochem.com (H.O.); yoshiyuki.ishida@cyclochem.com (Y.I.); keiji.terao@cyclochem.com (K.T.)

**Keywords:** Kuding tea, ursolic acid, mice, feeding study, herbal extract, safety

## Abstract

Kuding tea (KT) is a traditional Chinese beverage rich in plant bioactives that may exhibit various health benefits. However, little is known about the safety of KT extract (KTE) when consumed long term at high doses as a dietary supplement. Therefore, in this study, we investigated aspects of the safety of KTE. Male C57BL/6 mice were fed a high-fat, high-fructose, Western-type diet (control) supplemented with either 12.88% γ-cyclodextrin (γCD), 7.12% KTE (comprising 0.15% ursolic acid, UA) encapsulated in 12.88% γCD (KTE-γCD), or 0.15% UA over a 6-week experimental period. The dietary treatments did not affect food intake, body weight or body composition. However, treatment with KTE-γCD, but not γCD and UA, increased liver weight and hepatic fat accumulation, which was accompanied by increased hepatic PPARγ and CD36 mRNA levels. KTE-γCD treatment elevated plasma cholesterol and CYP7A1 mRNA and protein levels compared to those in control mice. KTE-γCD substantially increased the mRNA and protein levels of hepatic CYP3A and GSTA1, which are central to the detoxification of drugs and xenobiotics. Furthermore, we observed a moderate elevation in hepatic CYP3A (5-fold change) and GSTA1 (1.7-fold change) mRNA levels in UA-fed mice. In vitro data collected in HepG2 cells indicated a dose-dependent increase in hepatic cytotoxicity in response to KTE treatment, which may have been partly mediated by UA. Overall, the present data may contribute to the safety assessment of KTE and suggest that KTE encapsulated in γCD affects liver fat storage and the hepatic phase I and phase II responses in mice.

## 1. Introduction

Kuding tea extract (KTE) is a bitter infusion that has been widely used in traditional Chinese medicine (TCM) over many centuries [1]. It has been suggested that Kuding tea (KT) exhibits potential anti-inflammatory and antibacterial properties [2] and that KT relieves heat, cough and itching [3]. KT is used as a medicinal plant to manage hypertension, hyperlipidaemia and obesity [1]. Furthermore, it has been shown that low dietary KT concentrations ameliorated hepatic lipid accumulation induced by a high-fat diet in mice [4]. KT is rich in polyphenols and saponins, including kudinosides, as well as chlorogenic acids (CGAs), which may be partly responsible for its bitterness [1]. Other important constituents of KT comprise terpenoids, including the pentacyclic triterpenoid ursolic acid (UA) [1]. UA has been previously studied in the context of diet-induced non-alcoholic fatty liver disease (NAFLD) treatment [5,6,7].

The importance of herbal extracts in the nutraceutical industry is increasingly recognized. If herbal extracts, such as KT, are used as dietary supplements, their intake may substantially exceed their normal dietary consumption via food and beverages, such as tea [8]. The safety of plant extracts remains an unresolved issue and is often not systematically assessed prior to the entrance of herbal dietary supplements to the market [8]. To increase the stability, bioavailability and bioactivity of plant constituents, herbal extracts are often encapsulated. In this context, cyclodextrins (CDs), which are cyclic oligosaccharides [9], are widely used to improve not only the bioactivity of dietary supplements but also their sensory quality [9]. Plant bioactives frequently exhibit an astringent taste, thereby reducing consumer acceptance [10]. The bitterness of plant bioactives can be masked or suppressed through their inclusion into CDs by so-called guest-host chemistry [11]. Several studies in the literature have addressed the encapsulation of tea and its constituents in terms of potential health benefits. CD encapsulation elevated the antioxidant activity of green tea [12], and complexation of UA with hydrophilic CDs significantly increased its anti-proliferative activity in cultured cells in vitro [13]. Furthermore, encapsulated green tea extracts have been shown to improve bone quality in aged mice [14]. However, to the best of our knowledge, there are currently no data in terms of the safety of KTE encapsulated in γ-CD (KTE-γCD). Therefore, within this feeding study in laboratory mice, we addressed the question of whether a high KTE-γCD concentration affects liver fat metabolism and the expression of hepatic phase I and phase II enzymes, which are important in the detoxification of xenobiotics and drugs.

## 2. Materials and Methods

### 2.1. KTE-γCD-Complex Formation and UA Analysis 

**Figure 1:**
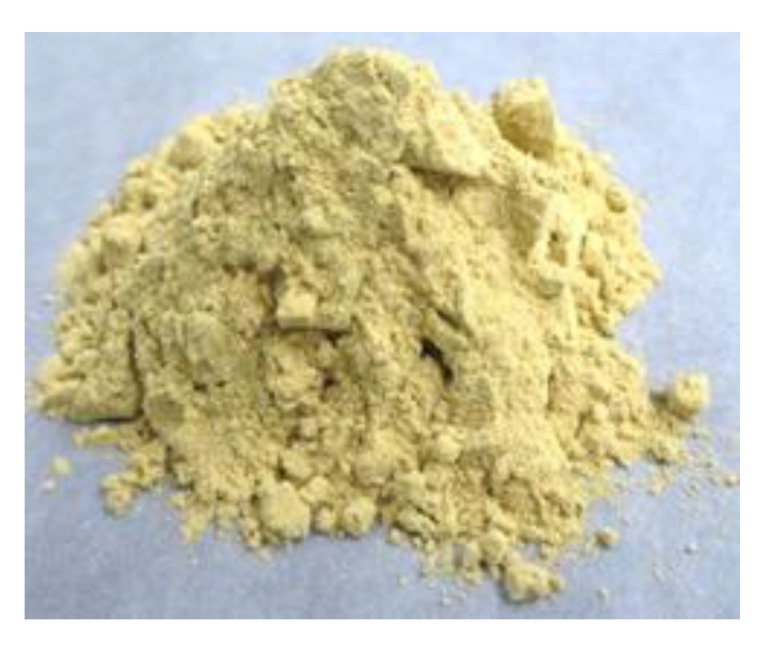
Kuding tea extract-γ-cyclodextrin (KTE)-complex used for the feeding study in mice.

To form the KTE-γCD-complex, large-leaved *Ilex kudingcha* (Japan Kampo NewMedicine Co., Ltd., Tokyo, Japan) was stirred in 60% ethanol for 1 h at 90 °C. After extraction, the leaves were removed by filtration, and ethanol was removed in vacuo. γCD was added to this extract (KTE: γCD = 1:2 *w*/*w*) and the solution was homogenized and dried by a spray dryer. The final KTE-γCD-complex used for the feeding study in mice is depicted in Figure 1. KTE was standardized in terms of its UA content. The concentration of UA in the KTE was analysed by HPLC using an Enduro C18 column (5 μm, 4.6 mm I.D. × 250 mm). The mobile phase consisted of acetonitrile:methanol:water: phosphoric acid at 50:40:10:0.5, and an isocratic profile with a flow rate of 1.0 mL/min was used at a temperature of 25 °C. UA (Tokyo Chemical Industry Co., Ltd., Tokyo, Japan) was used as an external standard. The UA stock solution (0.2 mg/mL) was prepared in methanol and filtered (Advantec Dismic-25, 0.2 μm). The KTE was dissolved in methanol. In the solution, UA was dissociated, and free UA was detected at 210 nm. The UA retention time was approximately 17 min, and the concentration of UA was calculated via an external standard curve using the peak area. The stability of UA in the KTE was evaluated by HPLC and the recovery was found to be 97% after storage for 6 weeks at 40 °C.

### 2.2. Mice and Diet

Fifty male C57BL/6NRj mice, aged 5 weeks, were purchased from Janvier Labs (Saint Berthevin Cedex, France). Mice were housed in groups (5 animals per cage) in Makrolon cages with environmental enrichment under controlled climatic conditions (22–24 °C, 55% relative humidity, 12 h light/dark cycle). The mice had free access to tap water and the experimental diets throughout the feeding study.

Mice were fed a purified, semisynthetic, energy-dense, high-fat, high-fructose diet (Ssniff S0065-E230) based on casein, corn starch and pork lard. The diet contained 23.1% crude fat, 17.7% crude protein, 16.5% starch, 20% fructose and 8.0% cellulose (metabolizable energy = 18.9 MJ/kg). After 2 weeks of adaption, the mice were divided by body weight into the following groups: control group (CON), γCD (CycloChem Bio Co., Ltd., Kobe, Japan) group, KTE-γCD (CycloChem Bio Co.) group or UA (Ark Pharm, Arlington Heights, USA) group (Table 1). The supplementation rates were chosen to provide equal amounts of UA in the UA-rich experimental diets based on a daily ingestion of approximately 150 mg of supplemental UA/kg of body weight.

Over the entire study period, the health conditions of the mice were controlled daily. Food intake and body weight were determined daily and weekly, respectively. We lost two animals over the entire time period, one before the intervention started and one from the γCD group. After 6 weeks on the experimental diets, body composition was measured. At the end of the trial, the mice were fasted for 5 h prior to euthanization with carbon dioxide. Blood was collected directly from the hearts. Tissue samples were removed, weighed, snap frozen and stored at −80 °C until further analysis. Samples for RNA isolation were stored in RNAlater (Qiagen, Hilden, Germany) and kept at −20 °C. Blood was also collected to obtain plasma and serum.

Animal studies were performed according to German and international regulations of animal welfare. The experimental protocol was approved by the local authority on 31 August 2018 (Ministry of Energy, Agriculture, the Environment, Nature and Digitalization, Schleswig-Holstein, V 242-28307/2018).

### 2.3. Determination of Body Composition

**Figure 2:**
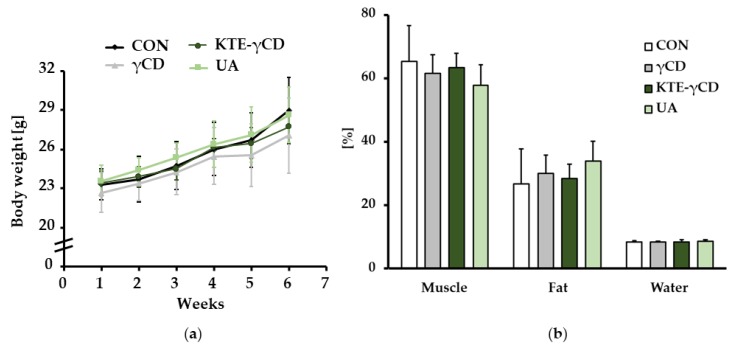
Effect of γ-cyclodextrin (ɣCD), Kuding tea extract-ɣCD (KTE-ɣCD) and ursolic acid (UA) on body weight development and body composition. Mice were fed (ad libitum) the following diets for 6 weeks: a high-fat, high-fructose, Western-type diet (CON, control), 12.88% γCD, 7.12% KTE encapsulated in 12.88% γCD (KTE-γCD) or 0.15% UA. (**a**) Body weight development and (**b**) body composition at the end of the experiment. Body composition was calculated in relation to total body weight. Data are expressed as the mean ± SD (*n* = 8–10 mice/diet). Statistical analyses of body weight development were carried out using the mixed factorial ANOVA method (factor time and diet) with repeated measures after checking for sphericity, followed by the Bonferroni post hoc test (time *p* < 0.001; diet *p* = 0.516; time*diet *p* = 0.305). Statistical analyses of body composition were performed using one-way ANOVA.

The body composition of the mice was measured by a Minispec LF90 nuclear magnetic resonance (NMR) analyser (Bruker Biospin MRI GmbH, Ettlingen, Germany, Figure 2). This analysis is a rapid, non-invasive examination of whole-body fat tissue, lean tissue and free fluid based on the response to various radiofrequency pulse sequences. Due to the short time of measurement and easy handling, there was no need to anaesthetize the mice, allowing the minimization of stress to the animals.

### 2.4. Cryostat Sectioning and Oil Red O Staining

Fresh liver was first incubated in 4% formaldehyde for 24 h at 4 °C and then stored in 1% paraformaldehyde at 4 °C. Cryostat sectioning was conducted as follows: frozen and gelatine- embedded tissue blocks were fixed with Tissue-Tek O.C.T. compound (Weckert Labortechnik, Germany) in the cutting chamber of a microtome (2800 Frigocut E, Reichert-Jung, Germany) and cut into 7 µm thick sections at −25 °C. Frozen sections on a microscope slide were stained with 3 g/L Oil Red O (ORO) and heamalaun (both Sigma-Aldrich Chemie GmbH, Darmstadt, Germany) and covered with Kaisers glycerol gelatine (Sigma-Aldrich). Liver samples were visualized with a light microscope (Leica Microsystems, Wetzlar, Germany; magnification 200× and 400×).

### 2.5. Blood Biochemical Analysis

Total plasma cholesterol and triacylglycerol were measured with commercially available colorimetric assays (Fluitest, Analyticon, Lichtenfels, Germany) according to the manufacturer’s protocol. The activity of hepatic transaminases alanine aminotransferase (ALT) in the serum was determined with a commercially available fluorometric assay kit (ALT Activity Assay Kit, Sigma-Aldrich) according to the manufacturer’s instructions.

### 2.6. RNA Isolation and One-Step Quantitative Reverse Transcription Real-Time Polymerase Chain Reaction

Total RNA was isolated from tissue stored in RNAlater (Qiagen) with peqGOLD TriFast (VWR International, Darmstadt, Germany) following the manufacturer’s instructions and as described previously [15]. A one-step quantitative reverse transcription real-time polymerase chain reaction (one-step qRT-PCR) was carried out with a SensiFAST SYBR No-ROX One-step Kit (Bioline, Luckenwalde, Germany) and SYBR Green detection according to the manufacturer’s protocol using a Rotor-Gene 6000 thermocycler (Corbett Research, Sydney, Australia). Primers were designed with Primer 3 Input software (version 4.1.0) and purchased from Eurofins MWG (Ebersberg, Germany). Corresponding primer sequences and annealing temperatures are given in Supplementary Table S1. RNA amplification for each sample was conducted in duplicate, and each run included a standard curve and a no-template control. Relative mRNA levels of target genes were normalized to 18sRNA gene expression, and fold changes relative to the control group (CON) are given.

### 2.7. Western Blot Analysis

Protein expression was determined in cytosolic lysates prepared from fresh liver tissue. Protein concentrations were determined with a Pierce bicinchoninic acid (BCA) Protein Assay Kit (Thermo Fisher Scientific, Bremen, Germany) according to the manufacturer’s instructions.

For Western blotting, samples containing 30 µg of protein each were heated with loading buffer, and a Western blot analysis was performed as previously described in detail [16]. Target proteins were identified using the corresponding primary (Supplementary Table S2) and secondary antibodies (1:4000 anti-rabbit; 1:3030 anti-goat). Immunoreactive proteins were detected with enhanced chemiluminescence (ECL) reagents (Thermo Fisher Scientific). Bands were visualized in a ChemiDoc XRS system (Bio-Rad, Munich, Germany). Target protein expression was related to the total protein load per lane.

### 2.8. Cell Culture Studies in HepG2 Hepatocytes

HepG2 cells (Institut für Angewandte Zellkultur, Munich, Germany) were cultured in RPMI medium (RPMI-1640, P04-18047, PAN Biotech GmbH, Aidenbach, Germany) supplemented with 10% fetal bovine serum (Thermo Fisher Scientific) and 1% penicillin/streptomycin (PAN Biotech GmbH). Cells were grown at 37 °C in a humidified 5% CO_2_ incubator in T75 or T175 flasks. Cytotoxicity was tested with a neutral red assay [17]. Therefore, HepG2 cells were seeded in 24-well plates and treated when at 80% confluence with the test compounds for 24 h. KTE (CycloChem Bio Co.; 0.06, 0.2, 0.6, 1.2, 2 mg/mL), UA (Ark Pharm; 3, 10, 30, 60, 100, 250 µM), chlorogenic acid (CGA; Sigma-Aldrich; 10, 50, 100, 250 µM) and kudinoside D (KudD; ChemFaces, China; 10, 50, 100, 250 µM) were dissolved in dimethyl sulfoxide (Carl Roth GmbH, Karlsruhe, Germany). The concentrations of KTE and UA were chosen to provide equal amounts of UA during treatment. In all cases, incubation was carried out in duplicate. Experiments were performed independently three times.

### 2.9. Pyrrolizidine Alkaloid Analysis of the KTE

The KTE was analysed for its pyrrolizidine alkaloid (PA) content (Institut Kirchhoff Berlin GmbH, Berlin-Mitte, Germany) using an online SPE-LC-MS/MS analysis system. In total, 28 PAs, which are relevant to teas and herbal teas according to the German Federal Institute of Risk Assessment (Bundesinstitut für Risikobewertung, Berlin, Germany), were determined.

### 2.10. Statistical Analysis

Data were analysed for normality of distribution (Kolmogorov–Smirnov and Shapiro–Wilk test). In the case of normally distributed data, the *t*-test or one-way analysis of variance (ANOVA) was applied, followed by a Scheffe post hoc test (hepatosomatic index) or, in the case of heterogeneous variances, the Games–Howell post hoc test (liver weight, SREBP1). In the absence of normally distributed data, the Mann–Whitney U test (MWU) was applied. Repeated measures (food intake, body weight) were analysed using a mixed factorial ANOVA (factor time and diet) after checking for sphericity followed by the Bonferroni post hoc test. The Greenhouse–Geisser adjustment was used to correct for violations of sphericity. The results were considered statistically significant at *p* < 0.05. IBM SPSS statistical software (version 25, IBM: Armonk, NY, USA) was used for all analyses.

## 3. Results

### 3.1. Feed Intake, Body Weight Gain, Body Composition, Liver Weight, Liver Fat Accumulation and Hepatic Steatosis

Mice completely refused the diet supplemented with KTE only. Therefore, this group had to be eliminated from the experimental design. Thus, we examined the effects of dietary γCD, KTE-γCD and UA in C57BL/6 mice fed a high-fat, high-fructose, Western-type diet over a 6-week experimental period. The average feed intake (2.45 ± 0.13 g) and body weight gain (Figure 2a) were not significantly different between the groups. Furthermore, body composition in terms of fat, muscle mass and water content remained largely unchanged in response to the different dietary treatments for 6 weeks (Figure 2b).

**Figure 3:**
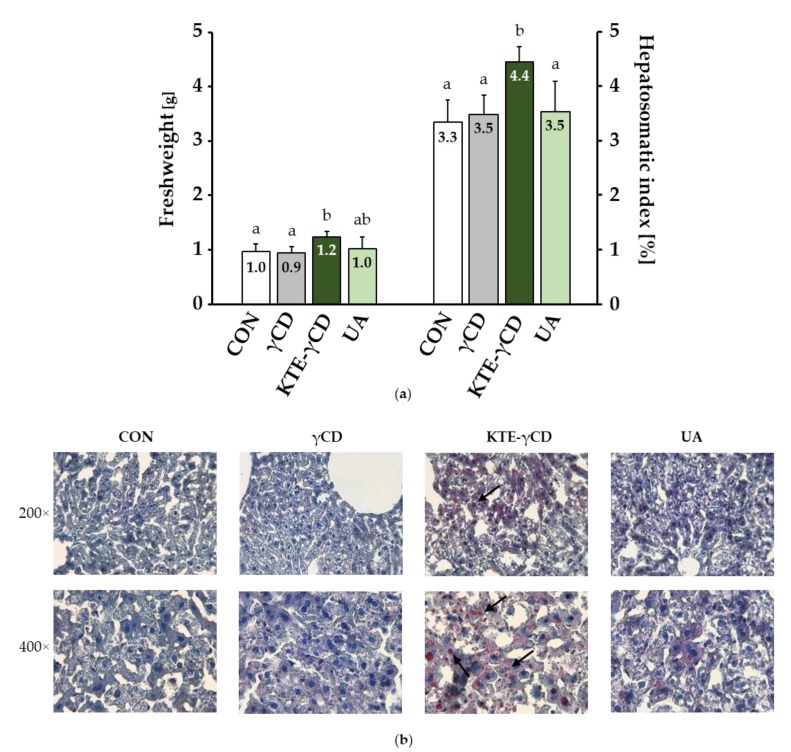
Kuding tea extract-γ-cyclodextrin (KTE-γCD) increased liver weight and hepatic fat accumulation. Mice were fed (ad libitum) the following diets for 6 weeks: high-fat, high-fructose, Western-type diet (CON, control), 12.88% γCD, 7.12% KTE encapsulated in 12.88% γCD (KTE-γCD) or 0.15% UA (UA). (**a**) The absolute liver weight and hepatosomatic index (liver weight/total body weight) were significantly increased in KTE-γCD-mice. Data are expressed as the mean + SD (*n* = 8–10 mice/diet). Statistical analyses of liver weight and hepatosomatic index were performed using one-way ANOVA. Different letters indicate statistically significant differences between treatments (*p* ≤ 0.05). (**b**) Representative liver histologic sections stained with Oil Red O (ORO) and haemalaun. Fat droplets in liver cells were stained red (arrows), and the nuclei were stained blue. Liver samples were visualized with a light microscope (magnification 200× and 400×).

Although no differences in body weight were observed between experimental groups, the absolute liver weight in mice fed KTE-γCD was significantly increased. Accordingly, the hepatosomatic index (liver weight/body weight) was significantly elevated in KTE-γCD-mice (Figure 3a). To clarify whether hepatic lipid accumulation was affected, cryostat sections were made, and liver tissues were stained with ORO. ORO staining revealed elevated lipid accumulation in hepatocytes from KTE-γCD-mice, suggesting fatty liver and hepatic steatosis (Figure 3b).

Hepatic steatosis is often accompanied by progressive inflammation, resulting in hepatic fibrosis [18]. Under the conditions investigated, serum ALT levels were not significantly different between the groups (CON, 15.5 ± 2.35 mU/mL; γCD, 16.2 ± 2.11 mU/mL; KTE-γCD, 17.3 ± 2.71 mU/mL; UA, 16.5 ± 2.67 mU/mL). Furthermore, mRNA levels of the inflammatory cytokine interleukin 1 beta (IL-1β) and the fibrogenic biomarker collagen, type I, alpha 1 (COL1A1) remained unchanged (Supplementary Table S3) in response to the different dietary treatments. Thus, there were no signs of hepatic fibrosis or cell injury in KTE-γCD-fed mice.

### 3.2. Hepatic Expression of Lipid Metabolism-Related Genes

**Figure 4:**
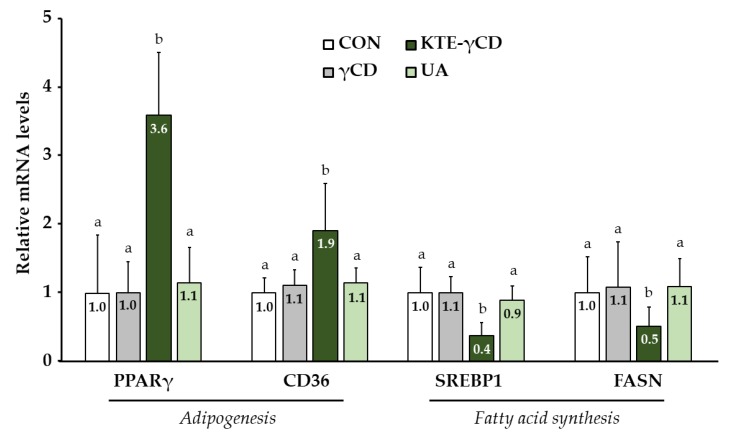
Kuding tea extract-γ-cyclodextrin (KTE-γCD) increased the mRNA levels of genes involved in adipogenesis (peroxisome proliferator activated receptor gamma, PPARγ; CD36 molecule, CD36), while lipid synthesis-related gene expression (sterol regulatory element binding transcription factor 1, SREBP1; fatty acid synthase, FASN) was decreased. Mice were fed the following diets ad libitum for 6 weeks: a high-fat, high-fructose, Western-type diet (CON, control), 12.88% γCD, 7.12% KTE encapsulated in 12.88% γCD (KTE-γCD) or 0.15% UA (UA). Gene expression was analysed via a one-step quantitative reverse transcription real-time polymerase chain reaction (one-step qRT-PCR). All qRT-PCR data were normalized to 18sRNA gene expression and are expressed in relation to the CON group. Data are given as the mean + SD (*n* = 8–10 mice/diet). Statistical analyses of SREBP1 mRNA levels were performed using one-way ANOVA (*p* ≤ 0.05). Significant differences in PPARγ, CD36 and FASN levels were calculated using the Mann–Whitney U test. Different letters indicate statistically differences between treatments (*p* ≤ 0.05).

To investigate whether hepatic lipid metabolism-related genes were influenced by dietary KTE-γCD, PCR measurements of selected genes encoding proteins involved in adipogenesis (peroxisome proliferator activated receptor gamma, PPARγ; CD36 molecule, CD36) and fatty acid synthesis (sterol regulatory element binding transcription factor 1, SREBP1; fatty acid synthase, FASN) were performed (Figure 4). Hepatic PPARγ and CD36 mRNA levels were upregulated by dietary KTE-γCD, while the mRNA levels of FASN and SREBP1 were decreased in response to dietary KTE-γCD supplementation compared to the three other groups (Figure 4).

### 3.3. Plasma Lipid Profile and Hepatic Cytochrome P450, Family 7, Subfamily A, Polypeptide 1 (CYP7A1)

**Figure 5:**
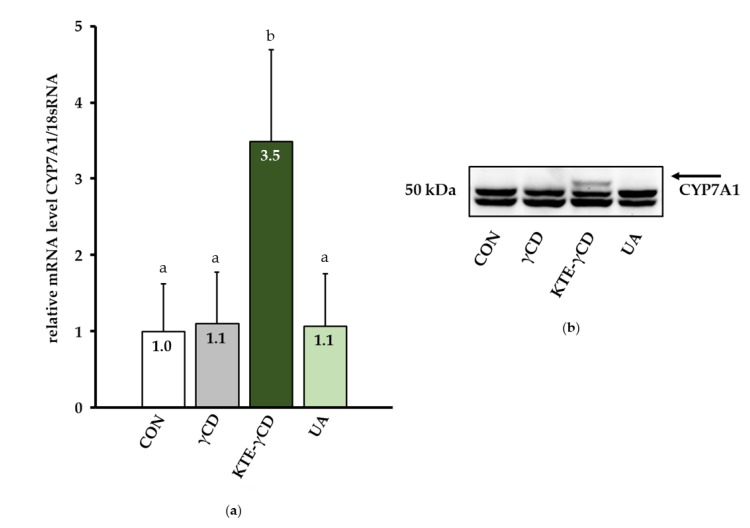
Kuding tea extract-γ-cyclodextrin (KTE-γCD) increased the mRNA (**a**) and protein (**b**) levels of cytochrome P450, family 7, subfamily a, polypeptide 1 (CYP7A1) compared to the other groups. Mice were fed the following diets ad libitum for 6 weeks: a high-fat, high-fructose, Western-type diet (CON, control), 12.88% γCD, 7.12% KTE encapsulated in 12.88% γCD (KTE-γCD) or 0.15% UA ursolic acid (UA). (**a**) Gene expression was analysed via a one-step quantitative reverse transcription real-time polymerase chain reaction (one-step qRT-PCR). The qRT-PCR data were normalized to 18sRNA gene expression and are expressed in relation to the CON group. Data are given as the mean + SD (*n* = 8–10 mice/diet). Statistical analyses of CYP7A1 mRNA levels were performed using the Mann–Whitney U test. Different letters indicate statistically significant differences between treatments (*p* ≤ 0.05). (**b**) Hepatic protein levels of CYP7A1 were determined by Western blotting, and a representative blot is shown. Representative bands from the stain-free UV image are shown in Supplementary Figure S1.

Biochemical analysis revealed that plasma total cholesterol levels were significantly higher in mice fed KTE-γCD than in all other experimental groups (CON, 194 ± 41 mg/dL; γCD, 214 ± 53 mg/dL; KTE-γCD, 457 ± 237 mg/dL; UA, 195 ± 36 mg/dL; *p* ≤ 0.05). Based on these data, we examined the mRNA and protein levels of cytochrome P450, family 7, subfamily a, polypeptide 1 (CYP7A1), which catalyses the first and rate-limiting step in bile acid synthesis when cholesterol levels are high. Both the mRNA (Figure 5a) and protein (Figure 5b) levels of CYP7A1 were higher in livers of mice fed KTE-γCD than in the other mice. However, plasma triacylglycerol levels remained unchanged in response to the different dietary treatments (CON, 100 ± 34 mg/dL; γCD, 84 ± 39 mg/dL; KTE-γCD, 97 ± 30 mg/dL; UA, 94 ± 30 mg/dL).

### 3.4. Phase I, Phase II and Phase III Metabolic Enzymes of Xenobiotic Biotransformation

**Figure 6:**
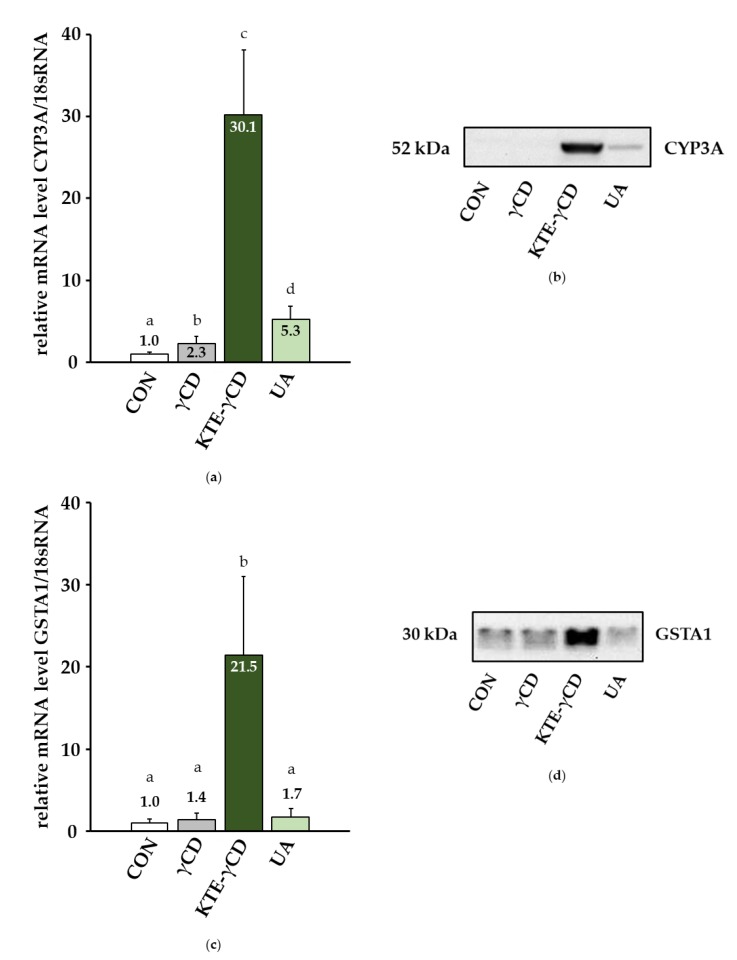
Effect of γ-cyclodextrin (γCD), Kuding tea extract-γ-cyclodextrin (KTE-γCD) and ursolic acid (UA) on the phase I and phase II enzymes of xenobiotic transformation in the mouse liver. Mice were fed the following diets ad libitum for 6 weeks: a high-fat, high-fructose, Western-type diet (CON, control), 12.88% γCD, 7.12% KTE encapsulated in 12.88% γCD (KTE-γCD) or 0.15% UA. Gene expression levels of cytochrome P450, family 3, subfamily a (CYP3A) (**a**) and glutathione S-transferase, alpha 1 (GSTA1) (**c**) were analysed via one-step quantitative reverse transcription real-time polymerase chain reaction (one-step qRT-PCR). All qRT-PCR data were normalized to 18sRNA gene expression and are expressed in relation to the CON group. Data are given as the mean + SD (*n* = 7–10 mice/diet). Statistical analyses of CYP3A and GSTA1 mRNA levels were performed using the Mann–Whitney U test. Different letters indicate statistically significant differences between treatments (*p* ≤ 0.05). Hepatic protein levels of CYP3A (**b**) and GSTA1 (**d**) were determined by Western blotting, and a representative blot is shown. Representative bands from the stain-free UV image are shown in Supplementary Figures S1 (CYP3A) and S2 (GSTA1).

Recent studies have proposed a link between xenobiotic exposure and the development of NAFLD, although the pathogenesis of NAFLD is multi-factorial [18]. To evaluate the effect of xenobiotic exposure, we investigated phase I, phase II and phase III enzymes in the livers of our mice. The mRNA levels of cytochrome P450, family 3, subfamily a (CYP3A) were increased more than 30-fold following dietary KTE-γCD supplementation and 5-fold following dietary UA supplementation for 6 weeks compared to controls (Figure 6a). Accordingly, the Western blot analysis indicated the substantial upregulation of CYP3A in the livers of KTE-γCD-treated mice and the moderate upregulation of hepatic CYP3A in UA-treated mice (Figure 6b). The mRNA and protein levels of the phase II enzyme glutathione S-transferase, alpha 1 (GSTA1) were also increased in KTE-γCD-treated mice compared to controls (Figure 6c,d). We observed the moderate induction of GSTA1 gene expression due to UA treatment (Figure 6c). Furthermore, γCD, KTE-γCD and UA treatment increased (1.68 ± 0.70, 6.51 ± 1.18 and 2.13 ± 0.72, respectively) the mRNA levels of phase III transporter ATP-binding cassette, subfamily C, member 3 (ABCC3) compared to the CON group (Mann–Whitney U test, *p* ≤ 0.05).

### 3.5. Cell Culture Studies in HepG2 Hepatocytes

**Figure 7:**
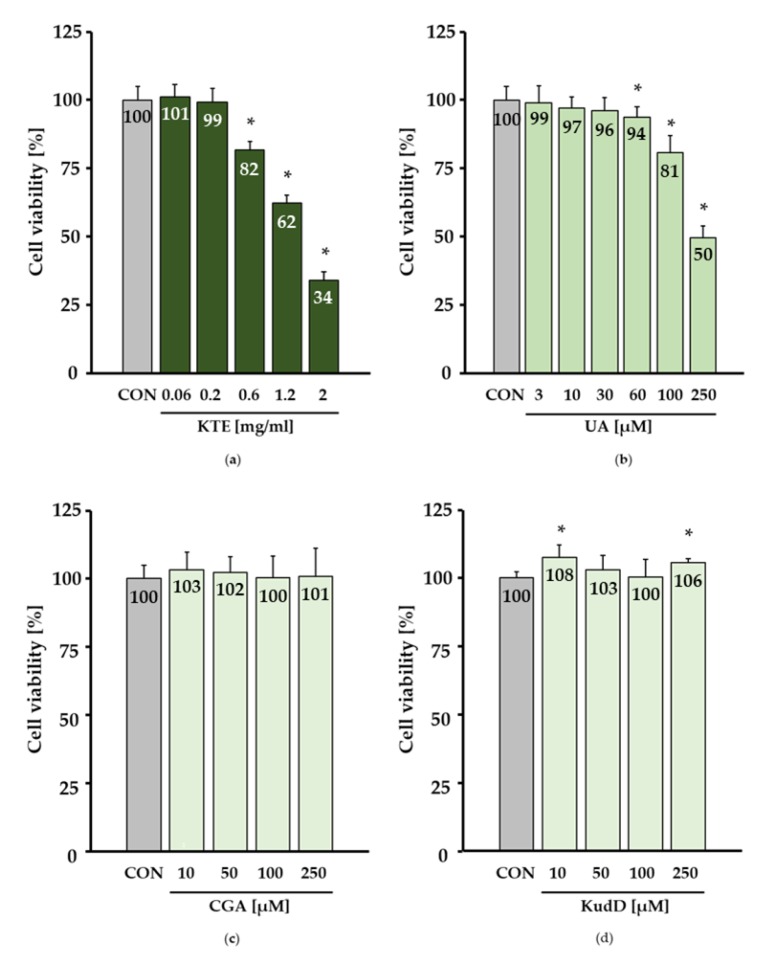
Dose-dependent increase in cytotoxicity due to Kuding tea extract (KTE) treatment in HepG2 hepatocytes (**a**). Ursolic acid (UA) at concentrations ≥ 100 µM exhibited moderate cytotoxicity in HepG2 cells (**b**). Cell viability remained unaffected in response to chlorogenic acid (CGA) (**c**) and kudinoside D (KudD) (**d**). Cell viability was measured via neutral red uptake assay. Cells were treated with KTE (0.06, 0.2, 0.6, 1.2, 2 mg/mL), UA (3, 10, 30, 60, 100, 250 µM), CGA (10, 50, 100, 250 µM) and KudD (10, 50, 100, 250 µM) for 24 h. All data are expressed in relation to the control group (CON, dimethyl sulfoxide). Data are given as the mean + SD. * indicates a significant difference compared with the CON group, analysed via a t-test or the Mann–Whitney U test (*p* ≤ 0.05).

In addition to in vivo studies in mice, we conducted in vitro studies in HepG2 cells. HepG2 cells were incubated with KTE (0.06, 0.2, 0.6, 1.2, 2 mg/mL), UA (3, 10, 30, 60, 100, 250 µM), CGA (10, 50, 100, 250 µM) and KudD (10, 50, 100, 250 µM) for 24 h. UA treatment was adjusted to match the UA concentration in the KTE. We observed a dose-dependent increase in cytotoxicity in response to KTE treatment (Figure 7a). Under the conditions investigated, pure UA (Figure 7b), but not CGA (Figure 7c) or KudD (Figure 7d), had a moderate cytotoxic effect on HepG2 cells.

### 3.6. PA Analysis of the KTE

PAs are highly hepatotoxic compounds that are widely distributed in plants, including herbal tea. Following their absorption, PAs undergo hepatic metabolic activation due to cytochrome P450-dependent enzymes [19]. For that reason, we analysed 28 different PAs in the KTE using a SPE-LC-MS/MS analysis. The concentrations of all the analysed PAs were below the limit of detection (5 µg/kg; Supplementary Table S4), indicating that the potential hepatotoxic effects of KTE are most likely not mediated by PAs.

## 4. Discussion

Approximately 20% of the US population consumes herbal dietary supplements [20]. Herbal dietary supplements, including KTE, are used for various reasons, including weight loss. Tea extracts consumed as a dietary supplement are often more concentrated than traditional tea infusions, as indicated by EFSA [21]. KT, a very popular beverage in China, is generally considered to be a safe food item [4]. In the present study, we administered a very high concentration (7.12%) of KTE. However, this concentration lies in a range similar to that of tea extract concentrations (1%–5%) used in other feeding studies [22,23,24,25]. Furthermore, we encapsulated KTE in γCD, which may have further increased the bioavailability of its constituents. Thus, the KTE concentration used within the present mouse study is beyond the range for its “normal” daily intake via KT consumption in the traditional way as a beverage. Mice completely refused the diet supplemented with KTE only, most likely due to its bitter taste and the high concentration we administered. The bitterness of KT has been previously reported [1]. However, to the best of our knowledge, complete refusal of KT-supplemented diets by mice has not yet been described in the literature.

It has been shown that low dietary KT concentrations prevents high-fat diet-induced obesity in mice [4]. However, we could not observe any anti-obesity activity of KTE in our study. Contrarily, we detected increased liver weight and hepatic fat accumulation. This could have possibly been caused by the high dietary KTE concentration as used in the present study. Weight loss products containing green tea extracts have been associated with liver injury [26], and the number of liver injury cases in the US related to herbal dietary supplements is increasing [27]. The underlying cellular and molecular mechanisms by which KTE affects liver metabolism have not yet been fully elucidated. In the present study, dietary KTE administration via a high-fat, high-fructose, Western-type diet over 6 weeks resulted in a fatty liver accompanied by the significant induction of CD36 and PPARγ. CD36 is a scavenger receptor central to the hepatic uptake of fatty acids and the pathogenesis of fatty liver disease [28]. Hepatic PPARγ was significantly elevated in patients with NAFLD, and PPARγ deletion in murine liver cells has been demonstrated to prevent liver injury and steatosis [29]. Thus, the induction of hepatic CD36 and PPARγ by KTE may have increased the storage of lipids in the livers of our mice. Accordingly, decreased steady-state mRNA levels of SREBP1 and FASN may be an adaptive response to counteract excess hepatic lipid storage in KTE-fed mice.

Studies in patients with steatosis, in vivo models in laboratory rodents, and cell experiments in lipid-overloaded cells suggested an association between increased lipid deposition and impaired CYP enzymes [30]. Furthermore, secondary plant bioactives and numerous drugs undergo extensive phase I and phase II metabolism, including activation due to cytochrome P450-dependent enzymes and glutathione conjugation via glutathione-S-transferases. In the present study, we observed the substantial induction of CYP3A and GSTA1 gene and protein expression due to dietary KTE and the moderate induction of CYP3A and GSTA1 gene and protein expression due to UA. Thus, herbal dietary supplements such as KTE and its constituents may interfere with drug metabolism, leading to altered drug bioavailability, increased toxicity and/or the loss of therapeutic efficacy, which warrants further investigation [31].

Dietary KTE administration was associated with elevated plasma cholesterol levels and the increased expression of hepatic CYP7A1, the key enzyme in bile acid synthesis. It was previously shown that CYP7A1 overexpression in mice plays an important role in protecting the liver against alcohol-induced steatohepatitis, whereas the lack of CYP7A1 induced hepatic inflammation [32]. Increased hepatic CYP7A1 expression, as observed in mice in response to KTE administration in the present study, may be an adaptive mechanism to protect against liver injury. Although KTE administration enhanced lipid storage in the liver and hepatic xenobiotic-metabolizing enzyme expression, inflammation- and steatosis-related gene expression as well as hepatic ALT activity remained largely unchanged in response to KTE treatment. These data thus suggest that KTE induced fatty liver but not hepatic fibrosis within the relatively short experimental feeding period of 6 weeks. Further studies are still required to examine potential hepatotoxicity of high dietary KTE supplementation and to decipher underlying cellular and molecular mechanisms more precisely.

Within this study, the constituents of KTE that increased liver fat accumulation and phase I and II responses in mouse livers in vivo were not fully clarified. Since UA at high concentrations induced cytotoxicity in HepG2 cells in vitro, the potential adverse hepatic effects of dietary KTE could have been partly attributed to UA. Importantly, we included an experimental group fed a diet supplemented with cyclodextrin only. In this group, liver fat and the expression of enzymes important in xenobiotic metabolism remained unchanged. Furthermore, we could not detect any PAs in the KTE. Hence, it is unlikely that PAs induced xenobiotic metabolism.

### Strengths and Limitations of This Study

A strength of this study was our connection of phenotypic data to molecular data concerning the potential toxicity of KT. Furthermore, we complemented mouse data collected in vivo with data in cultured cells collected in vitro. A limitation of our study was that we did not measure UA in the plasma of our mice. However, it should be noted that UA is cleared from the circulation relatively quickly [33], and the mice were fasted for 5 h prior to blood sampling. Likewise, we had only a very limited volume of plasma, which was also used for other analyses. Another limitation of our study was our exclusion of an UA-γCD group in the experimental design. Furthermore, we examined the effects of only a single concentration of dietary KTE over a relatively short experimental period. Therefore, additional feeding studies considering UA-γCD as well as a dietary KTE at a lower concentration fed over a longer time period should be undertaken.

## 5. Conclusions

In conclusion, the present data suggest that high dietary KTE supplementation induces fatty liver and increases hepatic xenobiotic-metabolizing enzymes in mice. Thus, our results contribute to the safety assessment of dietary KTE administered at a high concentration.

## Figures and Tables

**Table 1:** Experimental design.

Group	*n*	γCD (%)	KTE (%)	UA (%)
CON	10	---	---	---
γCD ^1^	10	12.88	---	---
KTE- γCD	10	12.88	7.12^2^	---
UA	10	---	---	0.15

^1^ Exchanged against corn starch. ^2^ Providing a supplementation of 0.15% UA.

## Supplementary Materials

The following Supplementary Materials are available online at https://www.mdpi.com/2072-6643/12/1/40/s1, Figure S1. Representative bands from the stain-free UV image to show protein loading of the CYP7A1 and CYP3A blot, Figure S2. Representative bands from the stain-free UV image to show protein loading of the GSTA1 blot, Table S1. Nucleotide sequences of primer used for one-step qRT-PCR, Table S2. List of primary antibodies used for Western blot, Table S3. Effect of γ-cyclodextrin (γCD), Kuding tea extract-γCD (KTE-γCD) and ursolic acid (UA) on mRNA levels of the inflammatory cytokine interleukin 1 beta (IL-1β) and the fibrogenic biomarker collagen, type I, alpha 1 (COL1A1), Table S4. Pyrrolizidine alkaloids (PAs) in Kuding tea extract (KTE).
