# Supplementary material for: High Dietary Kuding Tea Extract Supplementation Induces Hepatic Xenobiotic-Metabolizing Enzymes—A 6-Week Feeding Study in Mice
Source: Nutrients. 2019 Dec 22;12(1):40. doi: 10.3390/nu12010040 (PMC7019617; doi:10.3390/nu12010040)
Supplement: Supplementary file 1 [file nutrients-12-00040-s001.pdf]

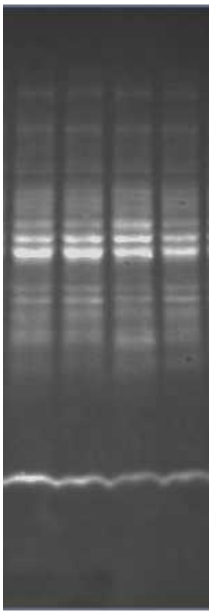

Figure S1. Representative bands from the stain-free UV image to show protein loading of the CYP7A1 and CYP3A blot.

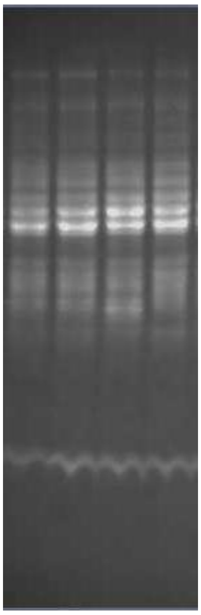

Figure S2. Representative bands from the stain-free UV image to show protein loading of the GSTA1 blot.

Table S1. Nucleotide sequences of primer used for one-step qRT-PCR.

Gene ID	Gene name	Symbol	Ta [°C]		Sequence
19791	*18s ribosomal RNA*	18sRNA	59	F<br>R	GGTAACCCGTTGAACCCCAT<br>CAACGCAAGCTTATGACCCG
76408	*ATP-binding cassette, subfamily C, member 3*	ABCC3	61	F<br>R	GTCCCCTGCATCTACCTGTG<br>GCCGTCTTGAGCCTGGATAA
12491	*CD36 molecule*	CD36	55	F<br>R	CAAAACGACTGCAGGTCAAC<br>CCAATGGTCCCAGTCTCATT
12842	*Collagen, type I, alpha 1*	COL1A1	58	F<br>R	TTCACCTACAGCACCCTTGT<br>AGTCCGAATTCCTGGTCTGG

13112	*Cytochrome P450, family 3, subfamily a*	CYP3A	55	F<br>R	CAAGGAGATGTTCCCTGTCA<br>CTGGTGATCACATCCATGCT
13122	*Cytochrome P450, family 7, subfamily a, polypeptide 1*	CYP7A1	55	F<br>R	TACAGAGTGCTGGCCAAGAG<br>GCTGTCCGGATATTCAAGGA
14104	*Fatty acid synthase*	FASN	59	F<br>R	GATGGAAGGCTGGGCTCTAT<br>TGCCTCTGAACCACTCACAC
14857	*Glutathione S-transferase, alpha 1*	GSTA1	58	F<br>R	TCACTACTTCAATGCCCGGG<br>GGGCACTTGGTCAAACATCA
16176	*Interleukin 1 beta*	IL-1β	55	F<br>R	CAGGCAGGCAGTATCACTCA<br>AGCTCATATGGGTCCGACAG
20787	*Sterol regulatory element binding transcription factor 1*	SREBP1	59	F<br>R	CCTAGAGCGAGCGTTGAACT<br>CAGAGAAACTGCAAGCAGGA
19016	*Peroxisome proliferator activated receptor gamma*	PPARγ	57	F<br>R	TGCCCAGATCTTCCTGAACT<br>TCTGTGAGAACCGCTAGCAA

Ta: annealing temperature; F: forward; R: reverse

Table S2. List of primary antibodies used for Western blot.

**Antibody**	**Company**	**Product number**	**Dilution**
CYP3A	Santa Cruz	sc-30612	1:500
CYP7A1	ABclonal	A10615	1:1000
GSTA1	Abcam	ab53940	1:2000

Table S3. Effect of γ-cyclodextrin (γCD), Kuding tea extract-γCD (KTE-γCD) and ursolic acid (UA) on mRNA levels of the inflammatory cytokine interleukin 1 beta (IL-1β) and the fibrogenic biomarker collagen, type I, alpha 1 (COL1A1). Mice were fed the following diets ad libitum for 6 weeks: a high-fat, high-fructose, Western-type diet (CON, control), 12.88% γCD, 7.12% KTE encapsulated in 12.88% γCD (KTE-γCD) or 0.15% UA. Gene expression levels of IL-1β and COL1A1 were analysed via one-step quantitative reverse transcription real-time polymerase chain reaction (one-step qRT-PCR). All qRT-PCR data were normalized to 18sRNA gene expression and are expressed in relation to the CON group. Data are given as the mean + SD (n = 8-10 mice/diet).

**Diet**	**IL-1β**	±	**SD**	**COL1A1**	±	**SD**
CON	1.00	±	*0.47*	1.00	±	*0.15*
γCD	1.27	±	*0.66*	1.27	±	*0.44*
KTE-γCD	0.97	±	*0.42*	1.29	±	*0.58*
UA	1.02	±	*0.38*	1.73	±	*1.17*

Table S4. Pyrrolizidine alkaloids (PAs) in Kuding tea extract (KTE).

**PA**	**Result**	**LOQ**[1]
Echimidine (Ech)	< LOQ	5 µg/kg
Echimidine-N-oxide	< LOQ	5 µg/kg
Erucifoline	< LOQ	5 µg/kg
Erucifoline-N-oxide	< LOQ	5 µg/kg
Europine	< LOQ	5 µg/kg

Europine-NOx	< LOQ	5 µg/kg
Heliotrine (Hel)	< LOQ	5 µg/kg
Heliotrine-NOx (Hel-NOx)	< LOQ	5 µg/kg
Intermedine	< LOQ	5 µg/kg
Intermedine-N-oxide	< LOQ	5 µg/kg
Jacobine	< LOQ	5 µg/kg
Jacobine-N-oxide	< LOQ	5 µg/kg
Lasiocarpine (Las)	< LOQ	5 µg/kg
Lasiocarpine-NOx (Las-NOx)	< LOQ	5 µg/kg
Lycopsamine (Lyc)	< LOQ	5 µg/kg
Lycopsamine-N-oxide	< LOQ	5 µg/kg
Monocrotaline	< LOQ	5 µg/kg
Monocrotaline-NOx	< LOQ	5 µg/kg
Retrorsine (Ret)	< LOQ	5 µg/kg
Retrorsine-NOx (Ret-NOx)	< LOQ	5 µg/kg
Senecionine (Snc)	< LOQ	5 µg/kg
Senecionine-NOx (Snc-NOx)	< LOQ	5 µg/kg
Seneciphylline (Snp)	< LOQ	5 µg/kg
Seneciphylline-NOx (Snp-NOx)	< LOQ	5 µg/kg
Senecivernine	< LOQ	5 µg/kg
Senecivernine-N-oxide	< LOQ	5 µg/kg
Senkirkine (Sen)	< LOQ	5 µg/kg
Trichodesmine	< LOQ	5 µg/kg

[1] LOQ = limit of quantification
